# Supplementary figures and images for: Fine Mapping of Five Grain Size QTLs Which Affect Grain Yield and Quality in Rice
Source: Int J Mol Sci. 2024 Apr 9;25(8):4149. doi: 10.3390/ijms25084149 (PMC11050437; doi:10.3390/ijms25084149)

**A**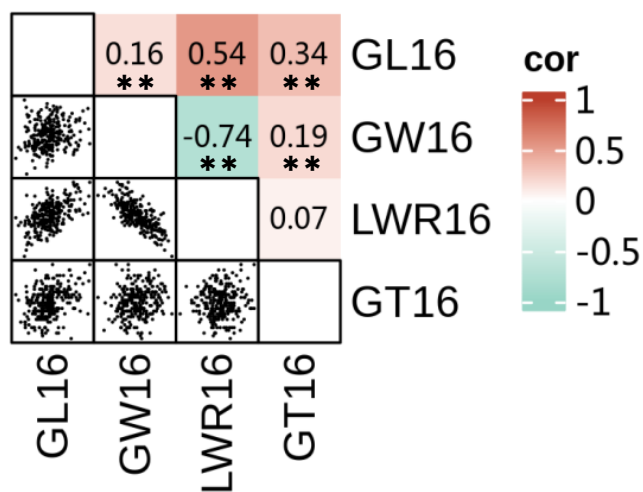**B**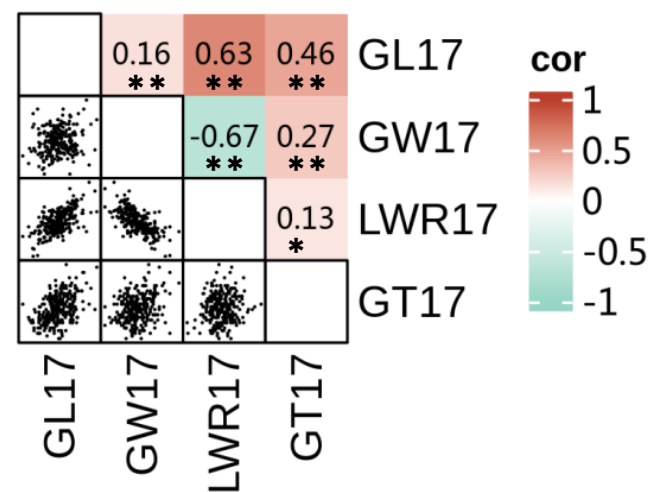**C**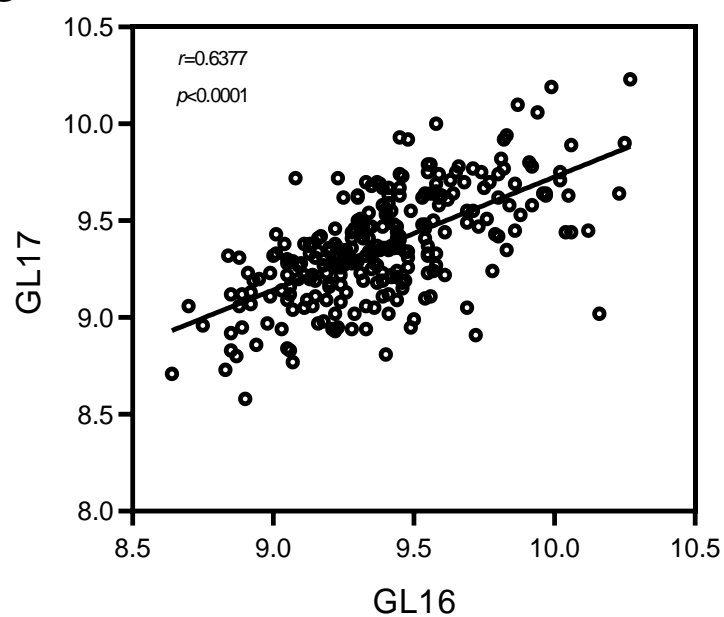**D**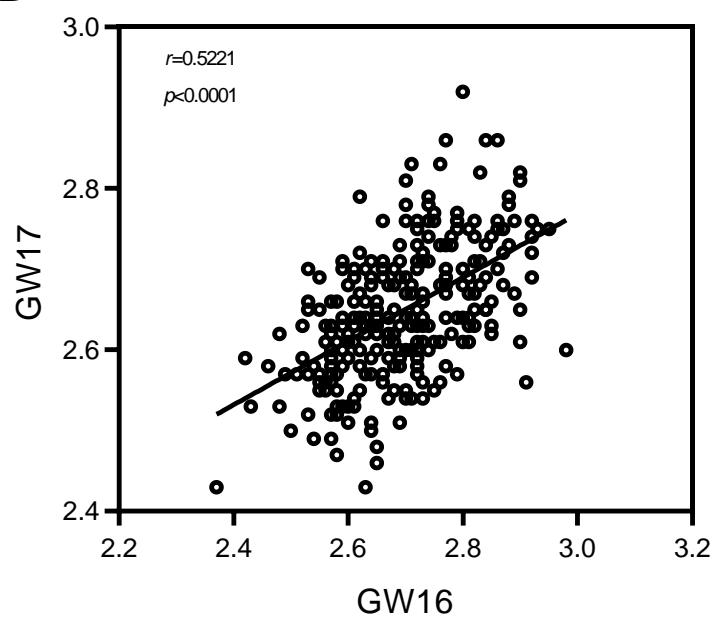**E**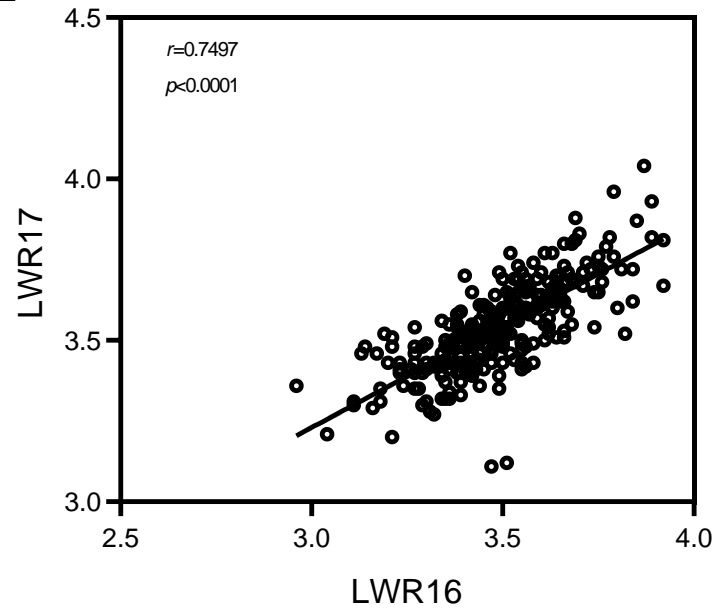**F**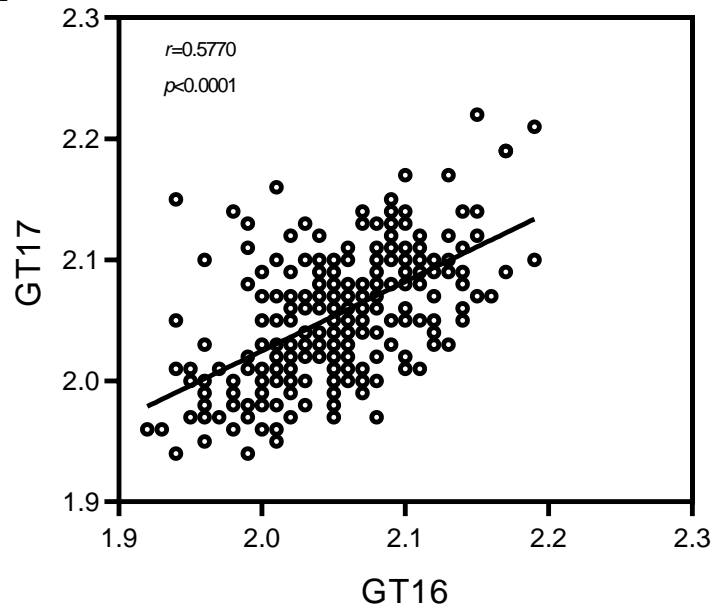

Supplement: Supplementary file 1 [file ijms-25-04149-s001.zip › Figure S1.pdf]

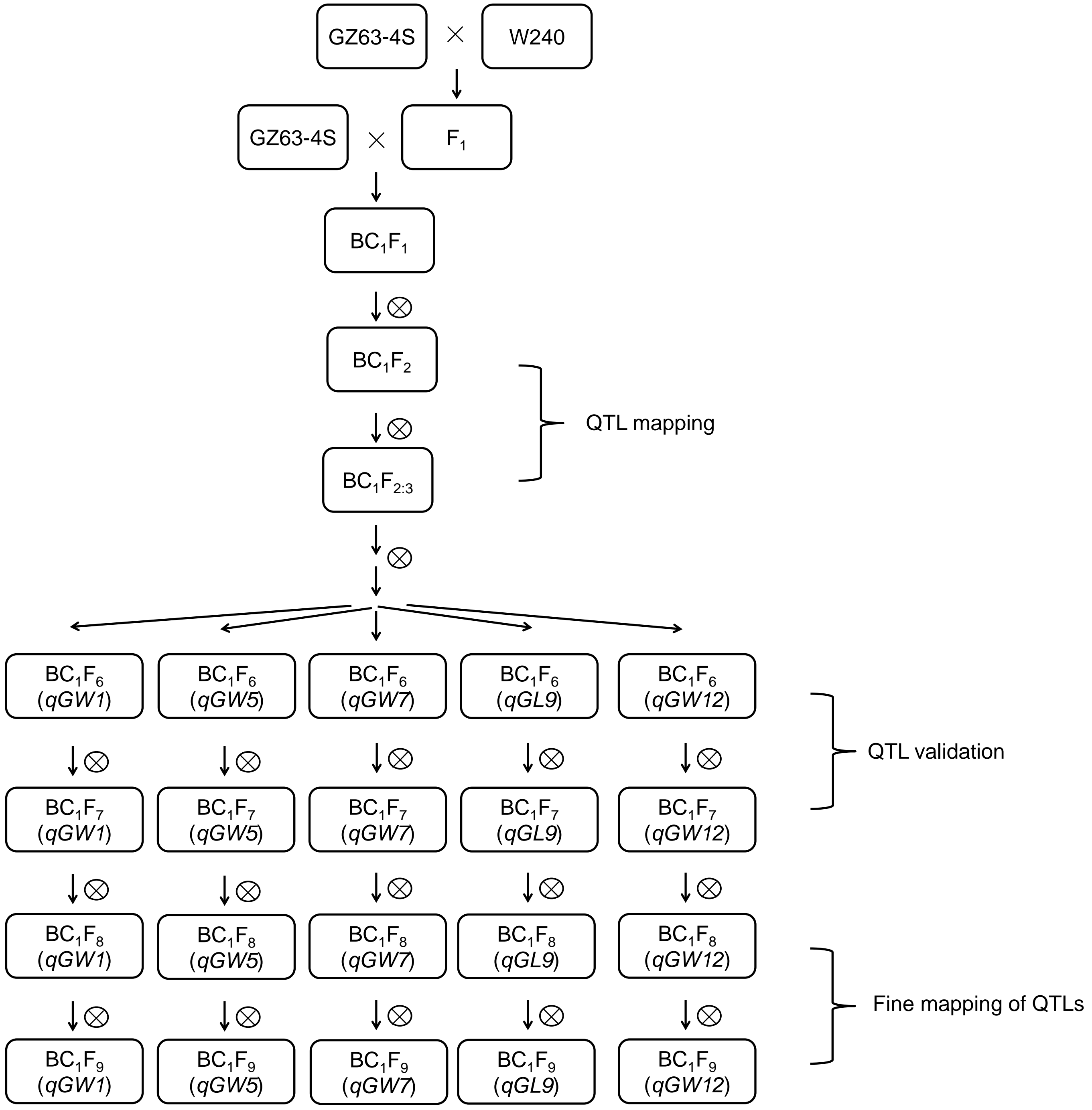

Supplement: Supplementary file 1 [file ijms-25-04149-s001.zip › Figure S2.pdf]

**A**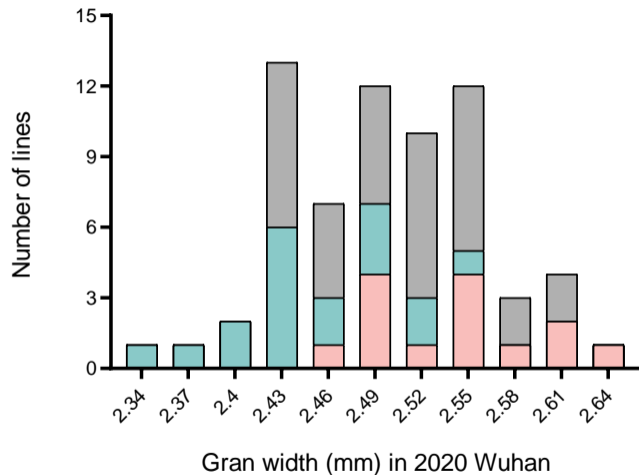**B**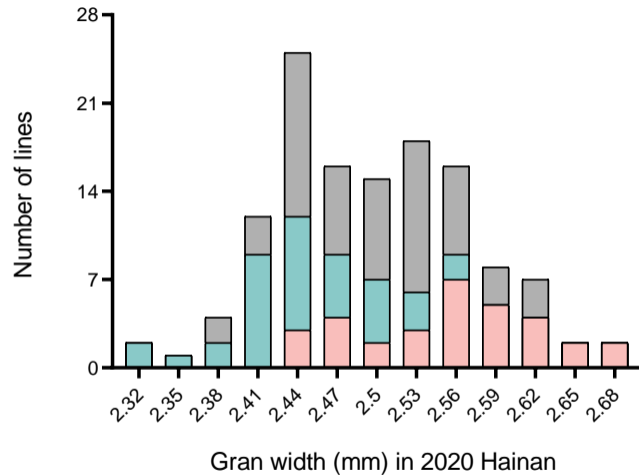

Homozygous W240

Homozygous GZ63-4S

Heterozygous

Supplement: Supplementary file 1 [file ijms-25-04149-s001.zip › Figure S3.pdf]

**A**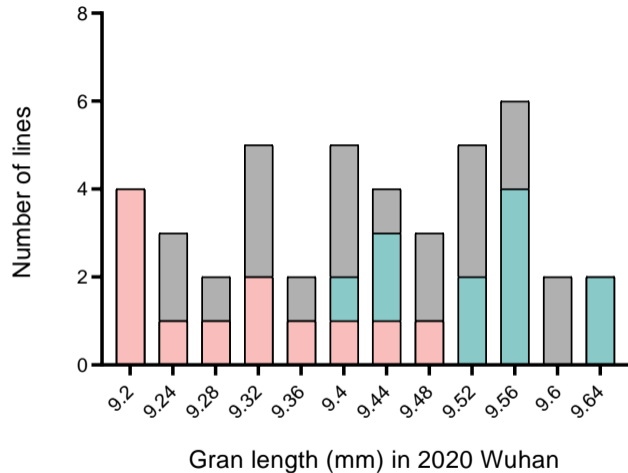**B**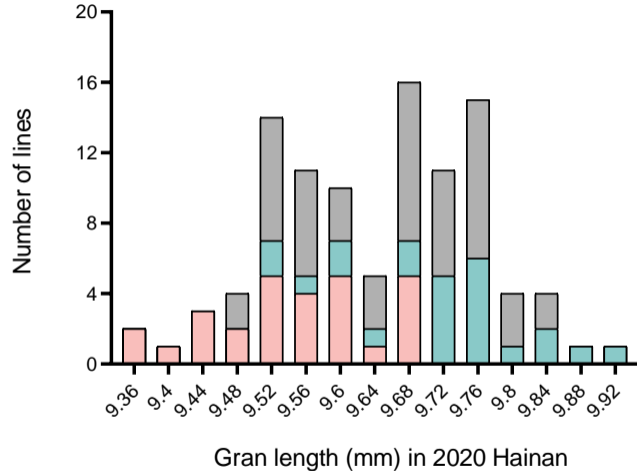

Homozygous W240

Homozygous GZ63-4S

Heterozygous

Supplement: Supplementary file 1 [file ijms-25-04149-s001.zip › Figure S4.pdf]

**A**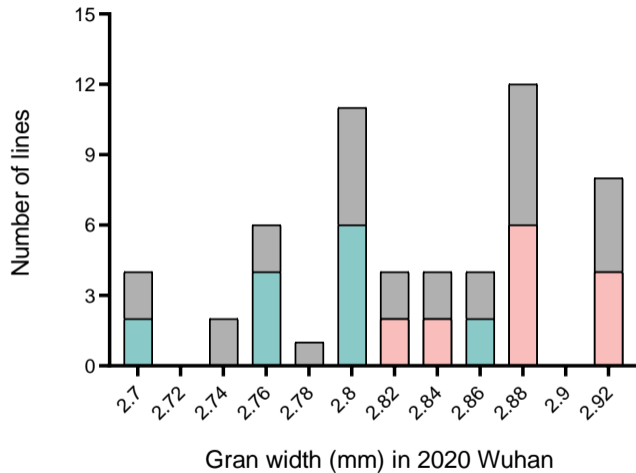**B**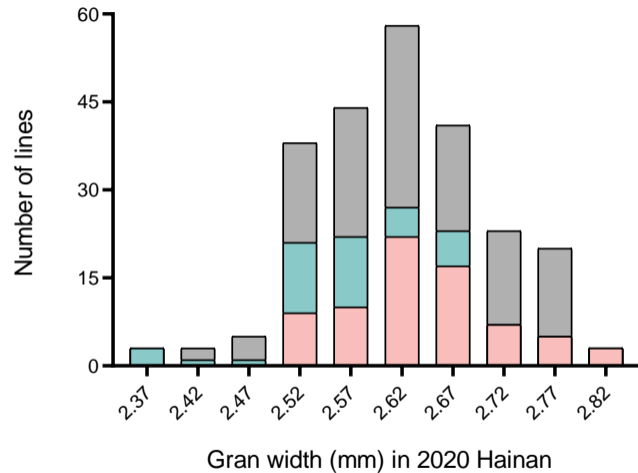

Homozygous W240

Homozygous GZ63-4S

Heterozygous

Supplement: Supplementary file 1 [file ijms-25-04149-s001.zip › Figure S5.pdf]

**A**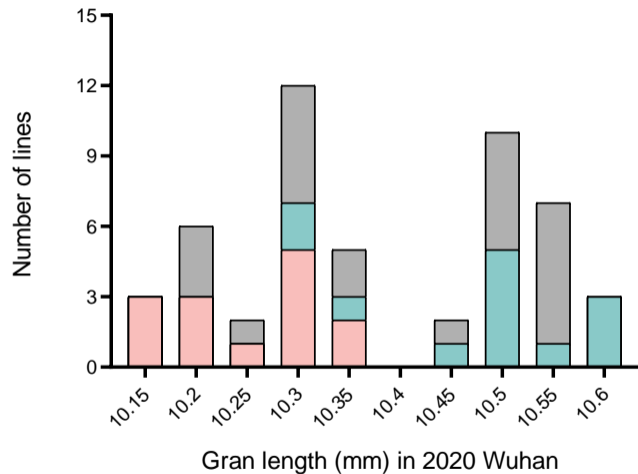**B**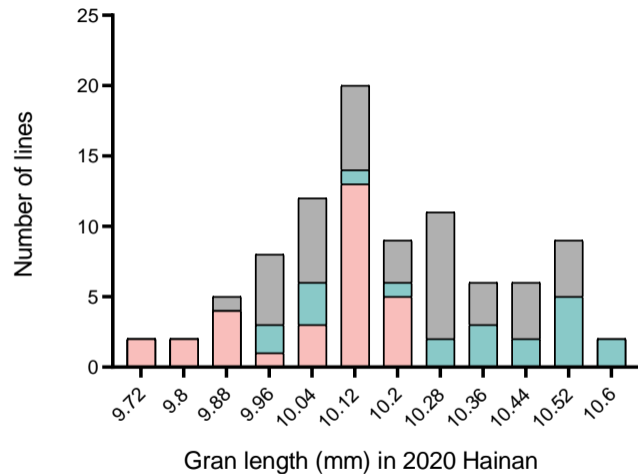

Homozoygous W240

Homozoygous GZ63-4S

Heterozygous

Supplement: Supplementary file 1 [file ijms-25-04149-s001.zip › Figure S6.pdf]

**A**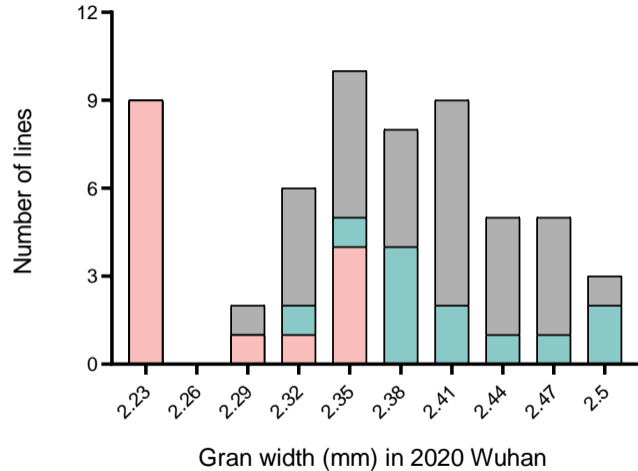**B**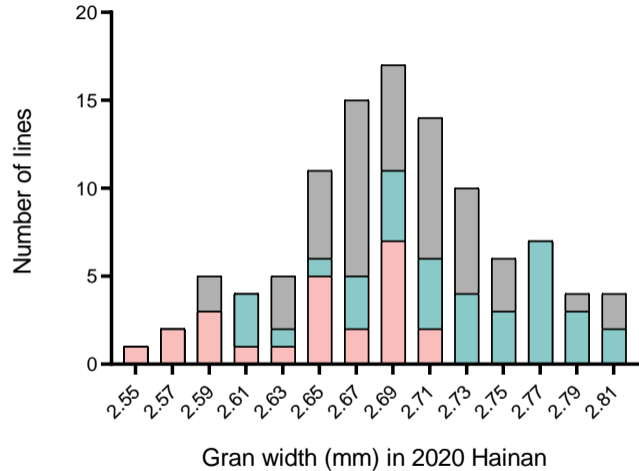

Homozygous W240

Homozygous GZ63-4S

Heterozygous

Supplement: Supplementary file 1 [file ijms-25-04149-s001.zip › Figure S7.pdf]
